# HIF-1α Is Essential for Effective PMN Bacterial Killing, Antimicrobial Peptide Production and Apoptosis in *Pseudomonas aeruginosa* Keratitis

**DOI:** 10.1371/journal.ppat.1003457

**Published:** 2013-07-18

**Authors:** Elizabeth A. Berger, Sharon A. McClellan, Kerry S. Vistisen, Linda D. Hazlett

**Affiliations:** Department of Anatomy and Cell Biology, Wayne State University School of Medicine, Detroit, Michigan, United States of America; Massachusetts General Hospital, Harvard Medical School, United States of America

## Abstract

Hypoxia-inducible factor (HIF)-1α, is a transcription factor that controls energy metabolism and angiogenesis under hypoxic conditions, and a potent regulator of innate immunity. The studies described herein examined the role of HIF-1α in disease resolution in BALB/c (resistant, cornea heals) mice after ocular infection with *Pseudomonas (P.) aeruginosa*. Furthermore, the current studies focused on the neutrophil (PMN), the predominant cell infiltrate in keratitis. Using both siRNA and an antagonist (17-DMAG), the role of HIF-1α was assessed in *P. aeruginosa*-infected BALB/c mice. Clinical score and slit lamp photography indicated HIF-1α inhibition exacerbated disease and corneal destruction. Real time RT-PCR, immunohistochemistry, ELISA, Greiss and MPO assays, bacterial load, intracellular killing, phagocytosis and apoptosis assays further tested the regulatory role of HIF-1α. Despite increased pro-inflammatory cytokine expression and increased MPO levels after knocking down HIF-1α expression, in vivo studies revealed a decrease in NO production and higher bacterial load. In vitro studies using PMN provided evidence that although inhibition of HIF-1α did not affect phagocytosis, both bacterial killing and apoptosis were significantly affected, as was production of antimicrobial peptides. Overall, data provide evidence that inhibition of HIF-1α converts a normally resistant disease response to susceptible (corneal thinning and perforation) after induction of bacterial keratitis. Although this inhibition does not appear to affect PMN transmigration or phagocytosis, both in vivo and in vitro approaches indicate that the transcriptional factor is essential for effective bacterial killing, apoptosis and antimicrobial peptide production.

## Author Summary

Infections of the eye, especially the cornea, often result in vision loss and may require corneal transplantation. *Pseudomonas aeruginosa* is a common, opportunistic bacterium that can infect the cornea, especially in extended wear contact lens users. There are more than 30 million such individuals in the US alone and they account for over 40% of corneal bacterial infection (keratitis) cases. The cornea itself is a specialized tissue that must effectively combat infections, while preserving intact visual acuity. Using various techniques to mimic in an animal model, what can occur in the human eye, we test how cells and molecules of the immune response react to the bacteria, leading to either its eradication or blindness. By better understanding the functions of the immune system during infection, rational design of more effective treatments for this disease, which preserve a patient's eyesight, are feasible. In addition, what we have learned about the different cells and molecules of the immune response in the eye may be applicable to other infectious diseases, as well.

## Introduction

While neutrophils (PMN) are essential for the effective clearance of microbial pathogens [1], they are functionally dependent on energetically intensive processes requiring ATP [2]–[4]. These events include production of toxic oxygen metabolites, triggering of respiratory burst, and biosynthesis of superoxide anions and other oxidizing agents such as hydrogen peroxide and formation of peroxynitrite [5], [6]; all of which contribute to effective bacterial killing. Hypoxic conditions, which are a well documented phenomenon of inflammation and infection [7]–[9], result in a decrease in both ATP and glucose. Despite a low oxygen environment, PMN (as well as other inflammatory cells) do not shift to mitochondrial respiration, but implement glycolysis as the primary metabolic pathway to generate ATP. It is essential that immune cells remain effective at cell migration, pro-inflammatory gene expression, phagocytosis and bacterial killing under such hypoxic conditions, which can be as low as <1% oxygen [10], [11]. Studies using glycolytic inhibitors have been shown to reduce not only cellular ATP concentrations, but also functional activity of myeloid cells [4]. As a hallmark characteristic, PMN are particularly reliant on the glycolytic pathway [12], [13] and uniquely adapted to hypoxic conditions on both metabolic and functional levels. In fact, a low oxygen microenvironment seemingly enhances the inflammatory response by immune cells [14] suggesting an alternate, yet highly functional hypoxic mode of existence [13].

The ability to remain functionally and metabolically competent under low O_2_ conditions is regulated by hypoxia-inducible factors (HIFs), which activate a hypoxic mode of adaptation [14]. HIFs are known to influence the immune response through regulation of cytokine expression, myeloid cell migration and other effector functions [14]. Low oxygen microenvironments associated with inflammation and infection have a marked effect on resident cells, as well as infiltrating inflammatory cells. These effects include adhesion, migration, and cell survival [11]. Further, recent studies have shown that bacterial exposure is a more potent stimulus for HIF-1α than hypoxia itself, and bacterial-induced HIF-1α stabilization readily occurs under normoxic conditions [15]. A number of comprehensive studies have focused on macrophages (Mφ, which express HIF-1α when activated. In regards to PMN however, it has been shown previously by Cramer et al. [13] that ATP levels were decreased ∼40% in HIF-1α null cells. Although these results indicate that HIF-1α is required for maintenance of intracellular energy homeostasis in PMN, the extent to which this cell type is functionally influenced by HIF-1α has yet to be examined. As such, the study described herein is the first to address the relationship of HIF-1α and PMN using a murine model of ocular (corneal) infectious disease. Experimentally and clinically, ocular infections of the cornea caused by *Pseudomonas* and other Gram-negative bacteria progress rapidly with considerable deleterious consequences [16]. Among these, coagulative necrosis surrounded by inflammatory epithelial edema and stromal ulceration are characteristic and can culminate in significant stromal tissue destruction and loss [16]. In addition, hypopyon (accumulation of leukocytes) and/or a ring infiltrate are often detected, along with descemetocele formation and corneal perforation, which is not uncommon [17]. Although Mφ are essential in the overall modulation of the immune response, PMN comprise up to 70% of circulating leukocytes in humans [18] (30–40% in mice [19], [20]) and combined with lymphocytes, constitute >85% of the infiltrated cells during inflammation. Parallel to clinical features, PMN are the major inflammatory cell transmigrating into the corneal stroma following infection in the mouse model [21]–[23]. The cell type is critical in both innate immunity and instructing acquired responses, as well as essential for effective uptake and killing of microbial pathogens. If not regulated appropriately, however, they induce extensive corneal “bystander” damage, leading us to examine how HIF-1α contributes to their ability to phagocytize and kill bacteria, produce antimicrobial peptides and undergo apoptosis.

## Results and Discussion

### Disease response after siRNA_HIF-1α_ treatment

**Figure 1:**
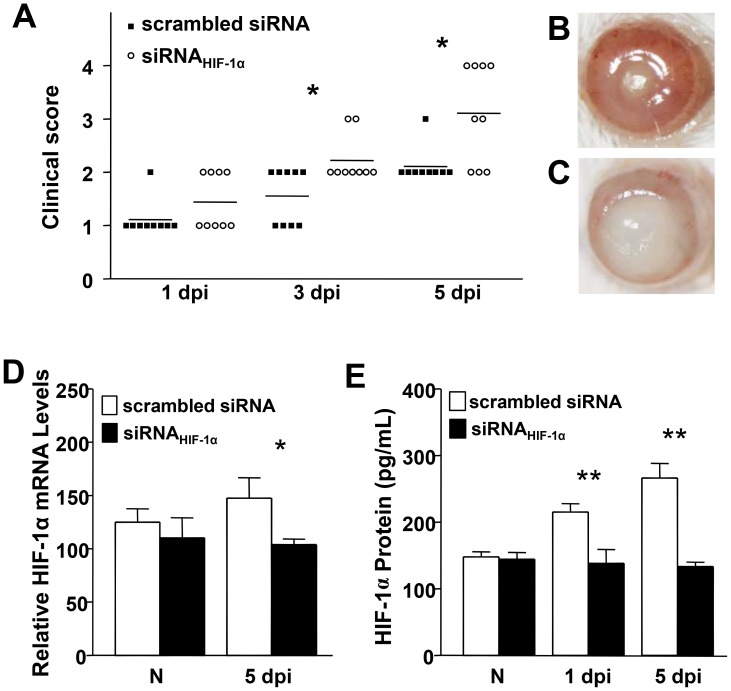
Disease response after siRNA_HIF-1α_ versus siRNA scrambled treatment. Clinical score (A) indicated statistically significant differences at 3 and 5 days p.i. between the two groups. Representative photographs taken by slit-lamp of *P. aeruginosa*-infected eyes at 5 days p.i. illustrated less disease (+2) in scrambled siRNA- (B) versus worsened disease (+3) in siRNA_HIF-1α_- (C) treated mice. Efficacy of HIF-1α silencing was confirmed at both mRNA (D) and protein (E) levels through 5 days p.i. Representative results from one of three independent experiments are illustrated. *P<0.05, ** *P*<0.01; slit lamp magnification = 8×.

When compared to the normal course of disease in BALB/c mice, knockdown of HIF-1α converted the characteristic resistant response to susceptible. This exacerbated disease response is demonstrated by clinical score (Fig. 1A), which was significantly higher at 3 and 5 days p.i. in siRNA_HIF-1α_ compared to scrambled siRNA treated controls. Disease score at 5 days p.i. was +3 (dense opacity, covering the entire anterior segment) with corneal perforation (+4) occurring in 40% of mice after siRNA_HIF-1α_ treatment versus lower scores (+2) and no perforation in the scrambled siRNA control mice. Photographs taken by slit lamp at 5 days p.i. (Fig. 1, B and C) illustrate the disease response in scrambled control (+2) (Fig. 1B) versus siRNA_HIF-1α_ treated mice (+3) (Fig. 1C).

**Figure 2:**
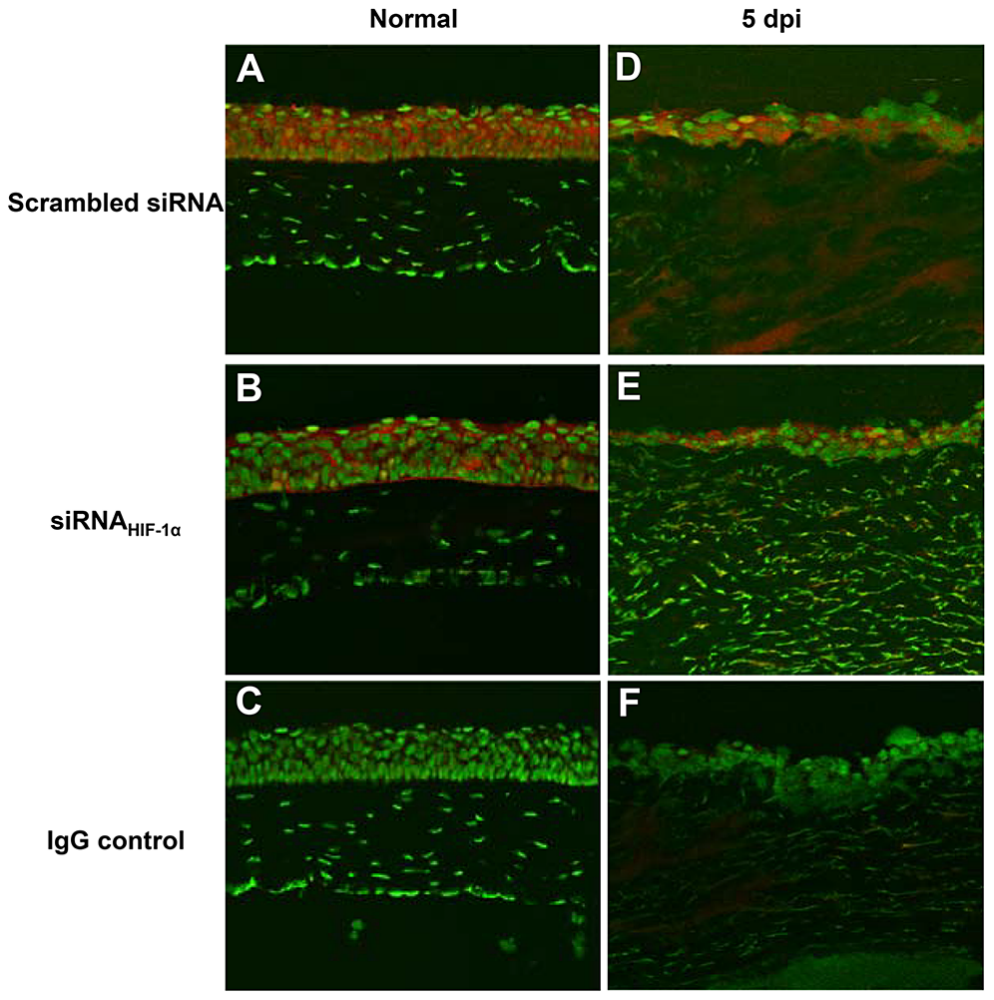
Immunostaining for HIF-α expression. Positive staining was detected in the epithelium of normal, uninfected corneas of both scrambled siRNA (A)- and siRNA_HIF-1α_-treated (B) mice. At 5 days p.i., increased HIF-1α expression was observed throughout the epithelium and stroma of scrambled siRNA-treated mice (D); while siRNA_HIF-1α_-treatment (E) revealed considerably less staining, indicating efficient knock-down of HIF-1α expression. IgG controls (C, F) were negative for staining. Representative results from two independent experiments (three mice per group per time point) are shown. Magnification = 185×.

Silencing of HIF-1α was assessed at both mRNA (Fig. 1D) and protein levels (Fig. 1E), and both analyses confirmed efficient gene knockdown. Further, immunostaining (Fig. 2) confirmed HIF-1α silencing in the normal (uninfected) eye and at 5 days p.i. In normal, uninfected, scrambled siRNA treated BALB/c mice (A), epithelial staining was of increased intensity compared with staining after siRNA_HIF-1α_ treatment (B). At 5 days p.i., both epithelial and stromal staining remained more intense in scrambled control (D) versus knockdown (E) treated mice. IgG controls were negative for HIF-1α staining (C and F).

### HIF-1α and cytokine/chemokine expression

**Figure 3:**
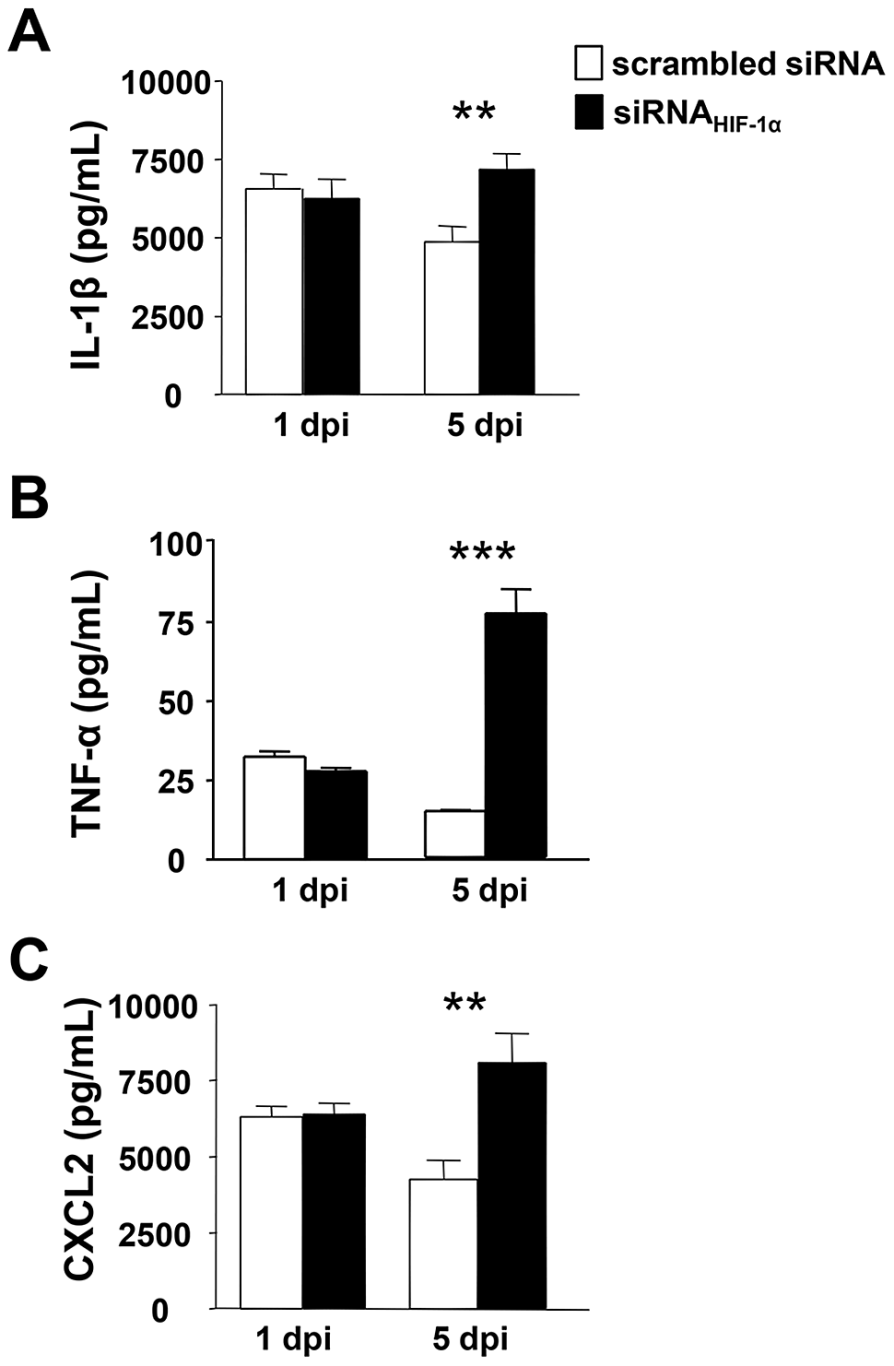
Pro-inflammatory cytokine/chemokine protein expression after HIF-1α silencing. Protein levels as detected by ELISA in scrambled siRNA- and siRNA_HIF-1α_-treated corneas at 1 and 5 days p.i. IL-1β (A), TNF-α (B) and CXCL2 (MIP-2) (C) levels showed no differences between groups at 1 day p.i. At 5 days p.i. however, all three molecules were significantly increased after HIF-1α silencing when compared to controls. Data represent two individual experiments each with five mice per group per time point. *P<0.05, ** *P*<0.01, *** *P*<0.001.

**Figure 4:**
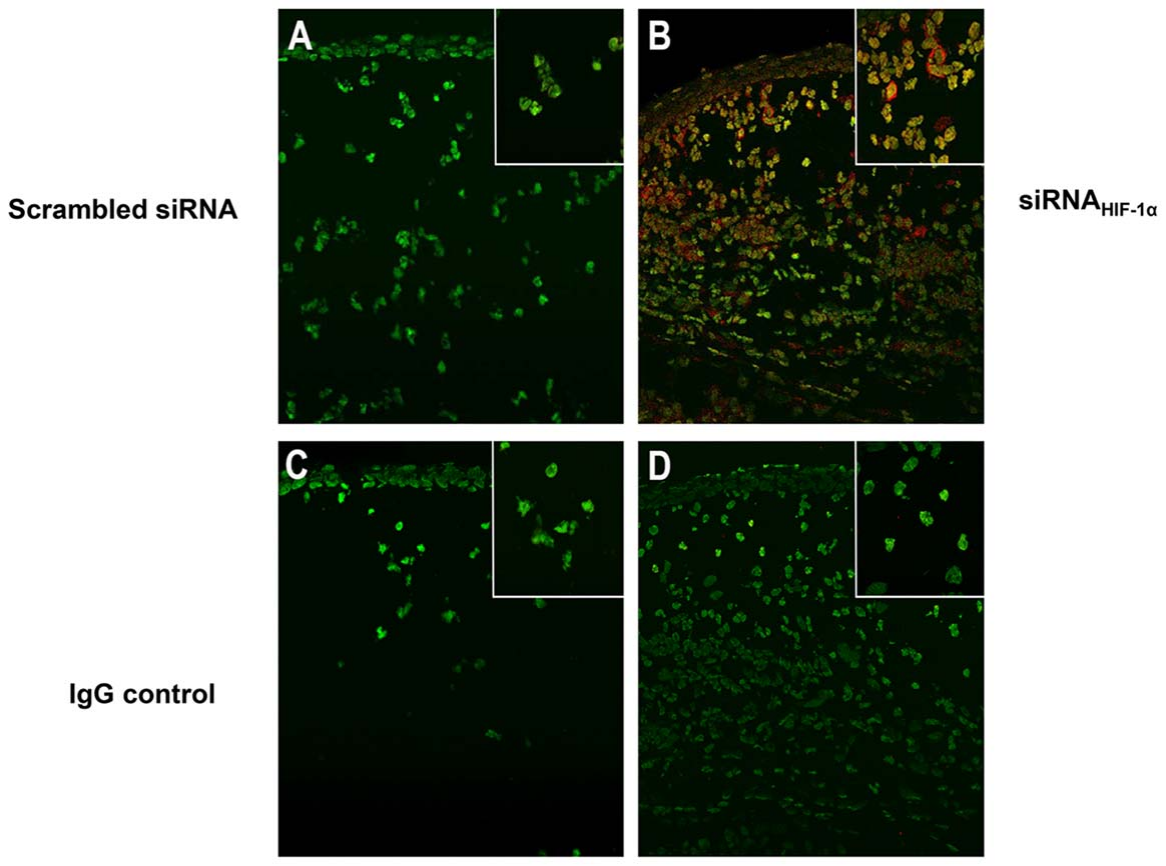
Immunostaining of T cell infiltrate after siRNA_HIF-1α_ treatment. No positive immunostaining for CD3ε, a pan T cell marker, was detected in the corneas of scrambled siRNA-treated mice (A) at 5 days p.i. In contrast, positive immunostaining (red) was observed in the peripheral cornea of HIF-1α silenced animals (B). The control sections shown in (C) and (D) were immunostained with species-specific IgG and were positive for SYTOX Green nuclear stain only. Images shown are representative of three independent experiments each with three mice per group. Magnification = 180×; inset = 335×.

Based upon these data, further studies tested whether loss of HIF-1α affected cytokine/chemokine expression, contributing to an impaired inflammatory response. Protein expression of select pro -inflammatory mediators known to be important in the host response to *P. aeruginosa*-induced ocular infection were analyzed at 1 and 5 days p.i. (Fig. 3). Results obtained from siRNA_HIF-1α_ treated mice revealed that IL-1β (A), TNF-α (B) and CXCL2 (MIP-2) (C) were significantly enhanced at 5 days p.i. when compared to scrambled controls. Previous in vivo work by Cramer et al. [13] using a phorbol ester TPA-induced ear inflammation model showed that loss of HIF-1α did not alter cytokine expression in resident myeloid cells. Though examined at the transcript level only, results from that study correspond with our mRNA (not shown) and protein data (both at 1 day p.i.). However, protein levels for these same molecules were significantly enhanced at 5 days p.i. after HIF-1α silencing, a time point which was not examined in the Cramer study. It is plausible that the increase in pro-inflammatory molecules at the later time point (5 days p.i.) may be due to infiltration of T lymphocytes as it has been shown that HIF-1α inhibition enhances T cell proliferation and pro-inflammatory cytokine production [24], [25]. In light of these data, we stained for CD3ε, a pan T cell marker, in corneas of siRNA_HIF-1α_ and scrambled siRNA treated mice at 5 days p.i. (Fig. 4A–D). Positive staining (red) in the peripheral cornea of siRNA_HIF-1α_ mice (A) indicated the presence of CD3^+^ T cells, while corneas of scrambled siRNA treated control mice were negative for CD3 staining (B) and appeared similar to IgG controls (C and D). These data also are consistent with past studies which demonstrated that T cells are not normally detected in the infected cornea of BALB/c mice [26] and that this may contribute to the resistance response. These results support the hypotheses that 1) HIF-1α regulates T cell infiltration after infection and 2) enhanced cytokine/chemokine production at 5 days p.i. is correlated with the presence of these cells.

### HIF-1α and the host inflammatory response

**Figure 5:**
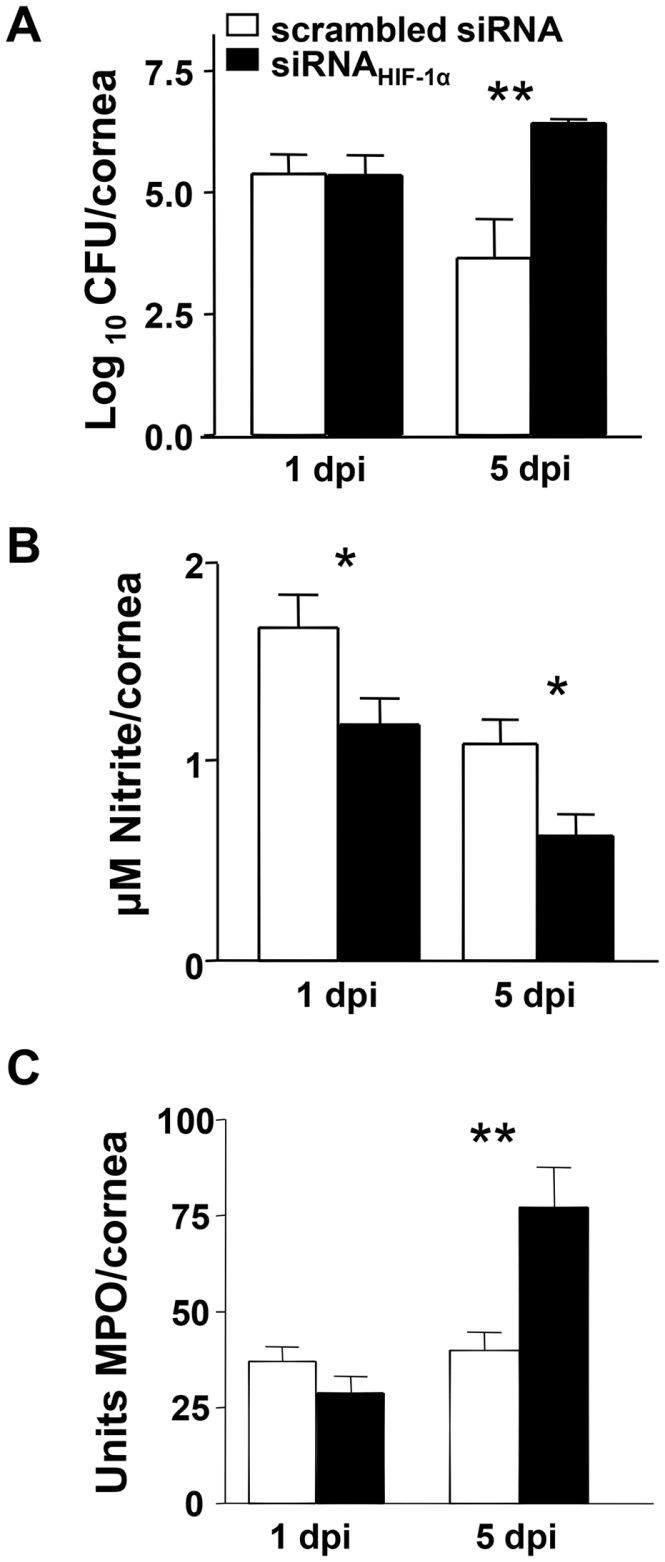
In vivo effects of siRNA_HIF-1α_ treatment. Bacterial counts (A) were detected from corneas of scrambled siRNA- and siRNA_HIF-1α_-treated mice after *P. aeruginosa* ocular infection. Results showed statistically more bacteria after HIF-1α silencing at 5 days p.i. Nitrite levels (B) were significantly reduced at both 1 and 5 days p.i. after siRNA_HIF-1α_ treatment when compared to scrambled controls. Corneal levels of MPO (C) were significantly increased at 5 days p.i. in siRNA_HIF-1α_ versus scrambled siRNA mice. Data represent three individual experiments each with five mice per group per time point. *P<0.05, ** *P*<0.01.

Bacterial load was measured to test whether HIF-1α knockdown impaired bacterial killing (Fig. 5A). At 1 day p.i., there was no difference in bacterial load between corneas of scrambled control and siRNA_HIF-1α_ treated mice. However at 5 days p.i., bacteria were decreased in the corneas of scrambled control versus knockdown treated mice, indicating that effective bacterial clearance was impaired. McInturff et al. [27] have shown that that HIF-1α protein expression was enhanced in areas of active NET formation in PMN. This group also showed that mammalian target of rapamycin (mTOR) is a post-transcriptional regulator of HIF-1α and furthermore, inhibition of mTOR and HIF-1 signaling in immune effector cells led to decreased bacterial killing. Interestingly, we have found that inhibition of mTOR by rapamycin in resistant BALB/c mice exacerbates disease after *P. aeruginosa*-induced ocular infection [28]. Therefore, we examined whether mTOR inhibition also decreased HIF-1α expression in our infection model as well, further implicating this molecule as an upstream regulator of HIF-1α. Results showed that corneal levels of HIF-1α mRNA were significantly reduced after mTOR inhibition compared to WT BALB/c mice at 1, 3 and 5 days p.i. (Fig. S1).

We next tested whether HIF-1α modulates either Mφ or PMN function during *P. aeruginosa*-induced ocular infection. During an inflammatory response, Mφ produce the free radical, nitric oxide (NO). As an indicator of Mφ activation, nitrite (a stable oxidized product of NO) was measured at 1 and 5 days p.i. (Fig. 5B). Corneas of siRNA_HIF-1α_ treated mice exhibited a significant decrease in nitrite levels at both time points when compared to scrambled controls. These results indicate that Mφ number and/or activity was reduced after HIF-1α silencing which correlates well with numerous studies investigating the effects of HIF-1α on this cell type [13], [15], [29]–[31]. For PMN, myeloperoxidase (MPO) was quantitated in the corneas of siRNA_HIF-1α_ and scrambled control treated animals at 1 and 5 days p.i. MPO is the most abundant protein in neutrophils, which (as a peroxidase enzyme) converts chloride and hydrogen peroxide into hypochlorous acid during the respiratory burst phase. MPO levels (Fig. 5C) did not differ between groups at 1 day p.i., however at 5 days p.i. there was significantly more MPO detected after siRNA_HIF-1α_ compared to control treated eyes. In contrast, using an in vivo ear inflammation model, Cramer demonstrated that loss of HIF-1α led to a decrease in MPO levels [13], but only a 24 h p.i. measurement was made. Zinkernagel et al. [32] used a murine model of *Staphylococcus aureus* skin infection and found no qualitative differences in the number of infiltrating PMN at the infection site after treatment with the HIF-1α inhibitor, mimosine; nor were there differences in respiratory-burst activity observed in PMN isolated from mimosine-treated mice. Thus, results regarding PMN infiltration appear to be inconsistent among studies; outcomes which are not solely determined by temporal factors, but also by inducing agent (e.g., infectious versus non-infectious) as well as tissue site (skin versus eye).

**Figure 6:**
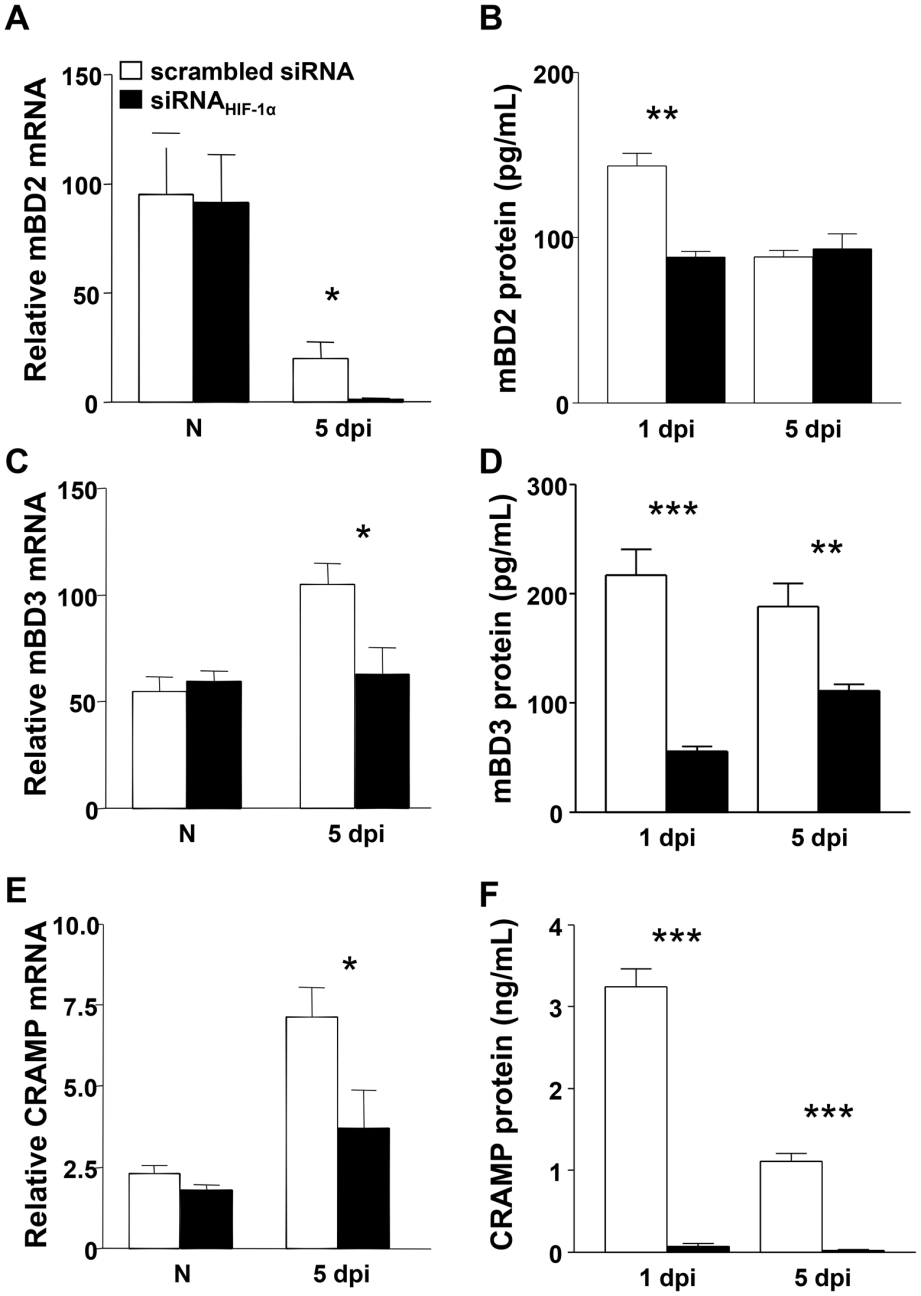
In vivo expression of antimicrobial peptides after HIF-1α silencing. siRNA_HIF-1α_ versus scrambled siRNA mice resulted in significantly decreased mRNA expression for mBD2 (A), mBD3 (C) and CRAMP (E) at 5 days p.i., while there were no differences between the two groups in normal, uninfected corneas. These data were confirmed at the protein level, indicating significant down-regulation of mBD2 (B), mBD3 (D) and CRAMP (F) at both 1 and 5 days p.i. after HIF-1α silencing compared to scrambled controls. Data represent two individual experiments with five mice per group per time point. *P<0.05, ** *P*<0.01, *** *P*<0.001.

Antimicrobial peptide production is a conserved component of the innate immune response. These peptides are broad spectrum antibiotics produced by corneal epithelial cells and host inflammatory cells, including PMN [33]–[35]. Peyssonnaux et al. [36] have shown that HIF-1α expression supports antibacterial function in keratinocytes through inducible cathelicidin production. Therefore, we next examined antimicrobial peptide expression in the corneas of siRNA_HIF-1α_ and scrambled control treated mice after infection. Corneal expression of murine beta defensin (mBD)2, mBD3 and cathelicidin-related antimicrobial peptide (CRAMP; LL-37 in humans), were examined under normal conditions and 5 days p.i. at the mRNA level and at 1 and 5 days p.i. for protein. Despite no differences in transcript levels in the normal (uninfected) cornea, all three peptides were significantly reduced after siRNA_HIF-1α_ treatment at 5 days p.i. (Fig. 6A, C and E). These results were confirmed at the protein level, which showed a significant decrease at both 1 and 5 days p.i. for mBD2 (B), mBD3 (D) and CRAMP (F) in siRNA_HIF-1α_ versus scrambled control treated mice.

### In vitro PMN studies

**Figure 7:**
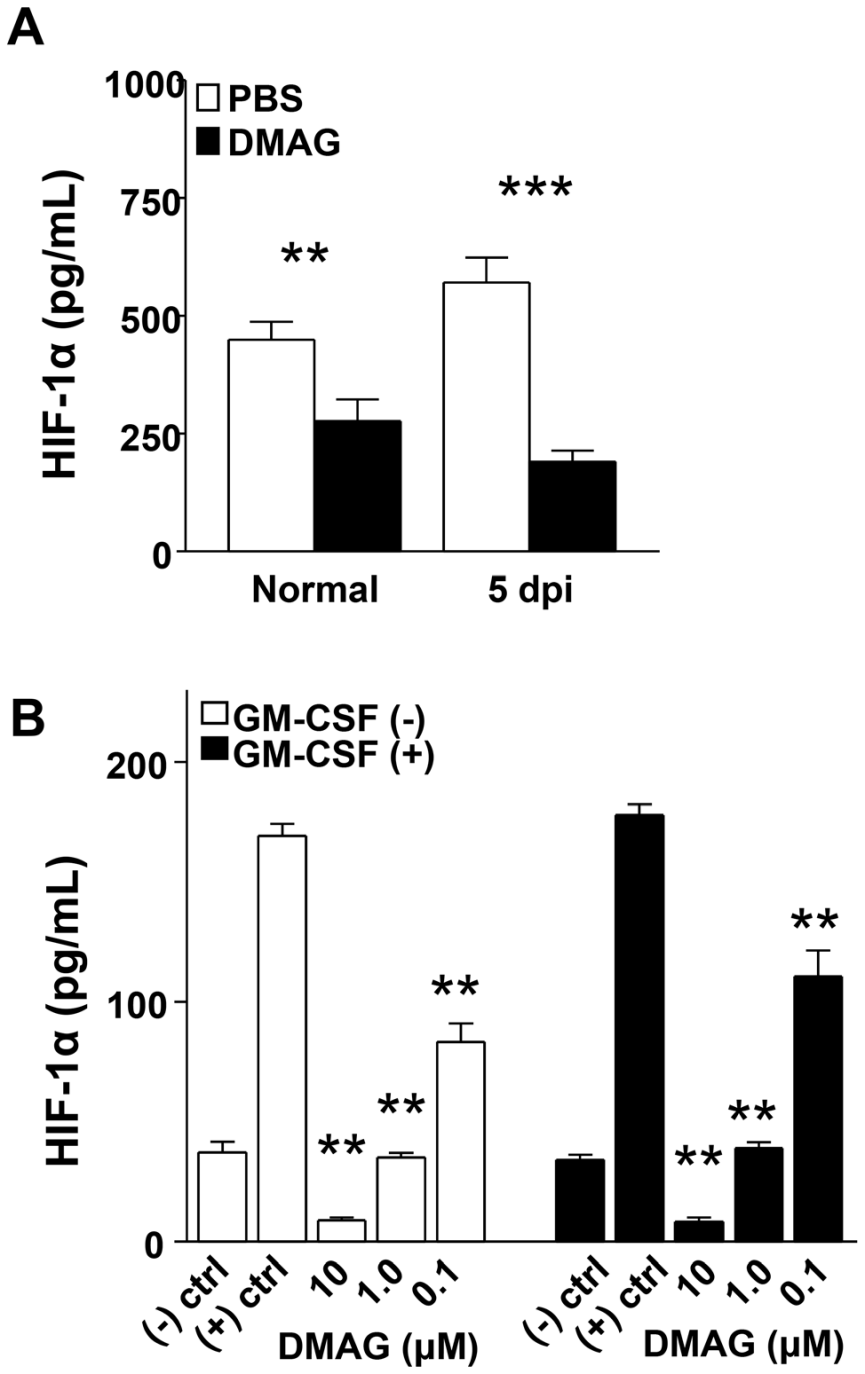
DMAG-induced inhibition of HIF-1α expression. As a complementary approach to siRNA_HIF-1α_ treatment, DMAG-induced inhibition of HIF-1α levels in vivo (A) was examined by ELISA in the normal (uninfected) cornea and at 5 days after ocular infection. HIF-1α protein was significantly reduced at both time points compared to PBS-treated controls. DMAG treatment was tested in vitro (B) using peritoneal-elicited PMN (+/− GM-CSF). Cells were stimulated with *P. aeruginosa* 19660 (2.5∶1 ratio of bacteria∶cells) +/− DMAG treatment (10, 1.0, 0.1 µM) for 18 hours, then HIF-1α protein levels from supernatants were determined by ELISA. Results showed that HIF-1α was significantly reduced after DMAG treatment at all three concentrations compared to positive controls (bacteria only). Negative control (no DMAG, no bacteria) showed baseline expression of HIF-1α protein. Data represent three independent experiments. *P<0.05, ** *P*<0.01, *** *P*<0.001.

PMN are the predominant cellular infiltrate in bacterial keratitis. Results obtained from in vivo studies indicated that PMN number was increased and/or function was enhanced after siRNA_HIF-1α_ treatment (as suggested by elevated MPO levels), however bacterial load was higher and disease response was more severe. Therefore, we next tested whether knockdown of HIF-1α impaired PMN phagocytosis and/or intracellular killing. For in vitro studies, we utilized the semi-synthetic chemical compound, 17-DMAG to inhibit HIF-1α as a separate, complimentary approach to siRNA treatment. We first tested 17-DMAG efficacy using the in vivo infection model, which revealed that HIF-1α protein expression was significantly reduced under normal conditions and at 5 days p.i. (Fig. 7A). Moreover, disease outcome (not shown) was similar to that observed after siRNA_HIF-1α_ treatment. Regarding in vitro treatment using 17-DMAG, PMN expression of HIF-1α protein was significantly down-regulated and was dose dependent (Fig. 7B). Given the shorter life span of PMN, we treated cells with or without granulocyte-macrophage colony-stimulating factor (GM-CSF), which prolongs cell survival and delays apoptosis [37], [38]. Therefore, all assays included both GM-CSF treated and untreated PMN to assess whether the effects observed were due to HIF-1α inhibition or decreased cell viability. With respect to HIF-1α inhibition by 17-DMAG, GM-CSF had no effect on HIF-1α expression levels when compared to untreated (no GM-CSF) cells (Fig. 7B).

**Figure 8:**
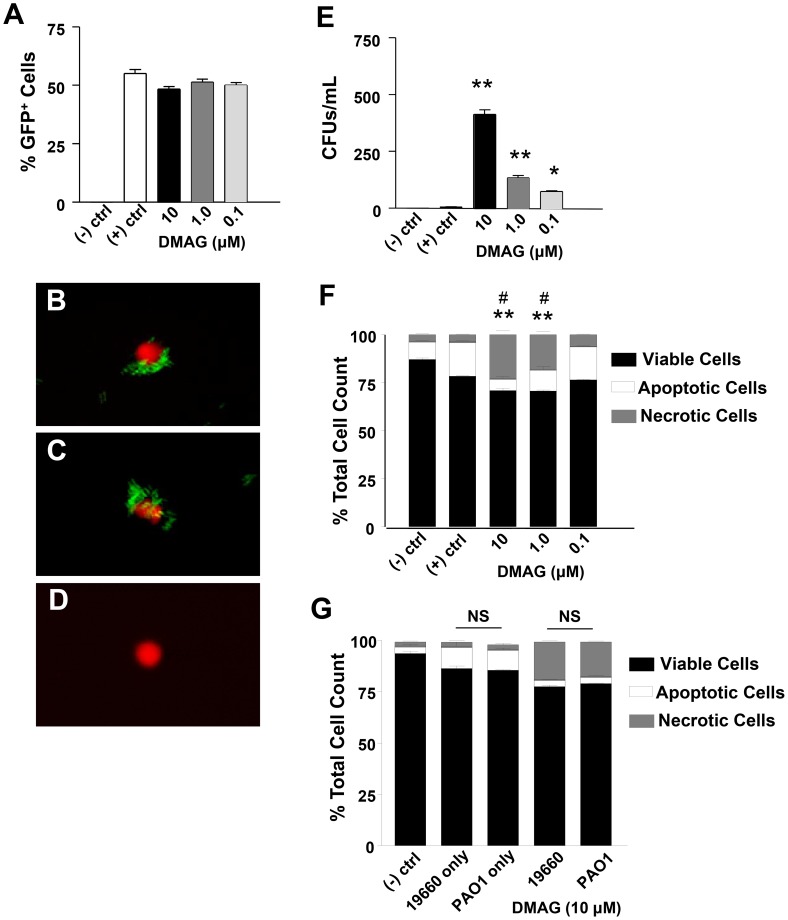
In vitro effects of HIF-1α inhibition on PMN function. Phaygocytosis (A), as measured by uptake of GFP^+^ *P. aeruginosa* 19660 by PMN, was not affected by DMAG-induced HIF-1α inhibition. Confocal laser scanning images documented intracellularly located GFP^+^ bacteria (green) with no DMAG treatment (positive control) (B) and after DMAG treatment (10 µM) (C). The negative control (no bacteria, no DMAG) (D) shows no GFP^+^ bacteria associated with the PMN, but stained positive for SYTOX Orange nuclear stain only. Intracellular killing by PMN (E) was analyzed by enumerating viable CFUs in the cell lysates. Significantly more viable bacteria were detected after DMAG treatment and affects were dose-dependent. Apoptotic and necrotic cells were measured (F) after HIF-1α inhibition and indicated that DMAG treatment significantly decreased apoptosis/increased cell necrosis in a dose-dependent response compared to positive controls (bacteria only, no DMAG). Apoptotic and necrotic cells were measured (G) after HIF-1α inhibition (10 µM) using both 19660 (a cytotoxic strain) and PAO1 (an invasive strain) and revealed no significant differences between cell viability, apoptosis and necrosis between the two bacterial strains. Data represent three independent experiments. *P<0.05, ** *P*<0.01 for apoptotic cell counts – (+) control versus DMAG treated; # *P*<0.01 for necrotic cell counts – (+) control versus DMAG treated. B, C, D magnification = 1,000×. NS = Not Significant.

In order to visually detect bacteria within actively phagocytizing PMN, green fluorescent protein labeled (GFP^+^) *P. aeruginosa* were employed. Results indicated no significant differences in bacterial uptake between normal (untreated) and 17-DMAG-treated cells (Fig. 8A). This was further documented visually using confocal laser scanning microscopy (Fig. 8B and C); thus, indicating that PMN phagocytosis of bacteria is not dependent on HIF-1α. Negative controls (no drug and no bacteria) were negative for GFP^+^ bacteria (Fig. 8D). Differences were observed, however, for intracellular killing, which is ATP-dependent [2]. In fact, a dose-dependent reduction of bactericidal activity was observed, whereby the highest concentration of HIF-1α inhibitor (10 µM 17-DMAG) resulted in over 400-fold more viable bacteria present within these cells when compared to positive controls (untreated cells incubated with bacteria only) (Fig. 8E). 17-DMAG at 1.0 µM and 0.1 µM resulted in approximately 100- and 70-fold more viable bacteria, respectively, when compared to untreated (bacteria only, positive controls). Cells that were not treated with drug, nor incubated with bacteria (negative control) had no growth. Similar trends were observed for phagocytosis and bacterial killing when cells were treated with GM-CSF indicating that the effects observed were due to HIF-1α inhibition versus cells dying in culture (data not shown).

PMN must undergo apoptosis as their clearance is essential for resolution of infection [39]. Therefore, we next measured cell viability and whether HIF-1α augmented apoptosis. Cell viability decreased after DMAG treatment when compared to negative controls (no bacteria, no inhibitor), yet remained comparable to cell viability for positive controls (bacteria only, no inhibitor) (Fig. 8F). A decrease in overall cell viability was expected given that the presence of bacteria will activate phagocytosis and killing by PMN; upon clearance of the microbe, these cells are programmed to undergo apoptosis so as to limit their ability to destroy other cells and tissue through prolonged activation and release of proteolytic enzymes [40]. Apoptosis was decreased (and necrosis increased) after DMAG treatment; and similar to phagocytosis and bacterial killing assays, a dose response was observed. DMAG treatment at the highest concentration (10 µM) resulted in the largest decrease in apoptosis/increase in necrosis (Fig. 8F). Similar trends were observed for cells treated with GM-CSF (data not shown). In general, decreased apoptosis suggests a failure to resolve active inflammation, whereby necrosis becomes more prevalent and ultimately contributes to tissue destruction.

Given that 19660 is a cytotoxic strain of *P. aeruginosa*, we sought to determine whether the observed decrease in apoptosis and increase in necrosis was, in fact, due to the release of toxic effector proteins such as ExoU [41]. As such, 19660 was compared to an invasive non-ExoU expressing strain of *P. aeruginosa* – PAO1 (ATCC 15692) [42]. Cell viability, apoptosis and necrosis of PMNs revealed no differences after exposure to either 19660 or PAO1 (Fig. 8G). After HIF-1α inhibition, there was a slight increase in necrotic cells after 19660 versus PAO1 exposure. Although insignificant, we suspect that this difference may be due to the prolonged presence of cytotoxins associated with 19660, which are not appropriately cleared/de-activated in a timely manner without an active HIF-1α pathway.

**Figure 9:**
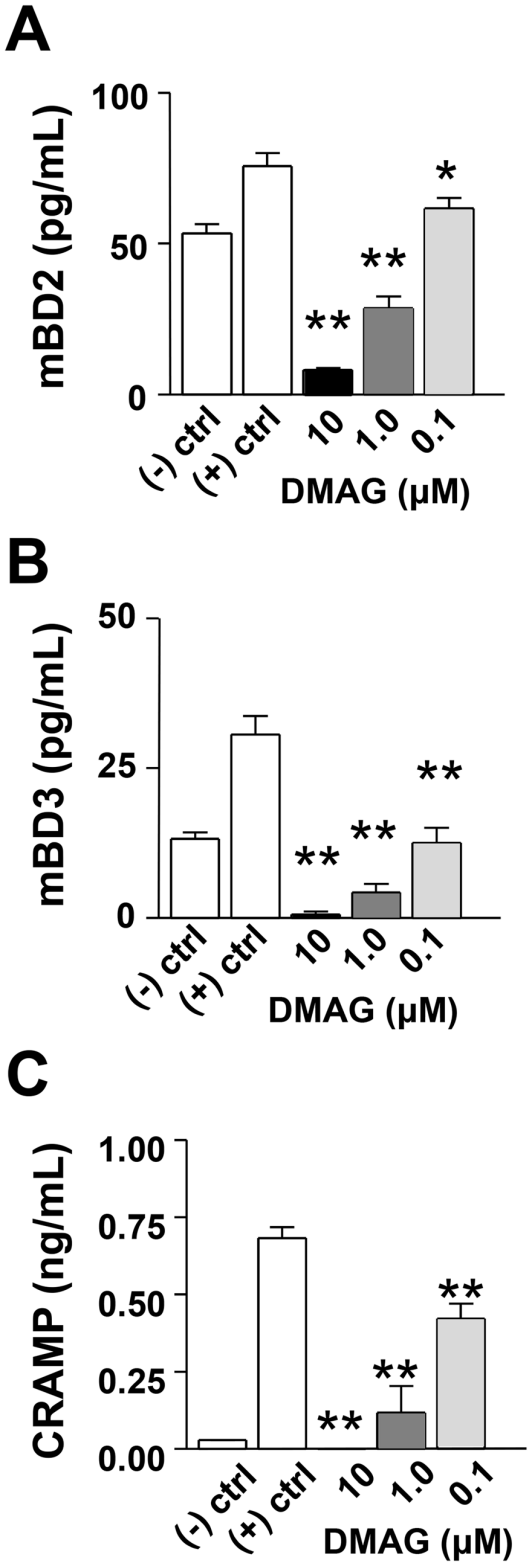
In vitro expression of antimicrobial peptides after HIF-1α inhibition. PMN were stimulated with *P. aeruginosa* +/− DMAG treatment (10, 1.0, 0.1 µM) for 18 hours, and antimicrobial protein levels from supernatants were determined by ELISA. mBD2 (A), mBD3 (B) and CRAMP (C) were significantly reduced in a dose-dependent response to DMAG treatment when compared to positive controls (bacteria only, no DMAG). *P<0.05, ** *P*<0.01.

Production of antimicrobial peptides by PMN contributes to effective removal of bacteria by this inflammatory cell. In fact, HIF-1α induces production of antimicrobial peptides by PMN [15], which are key effectors of killing (along with granule proteases) within NETs [43], [44]. Levels of mBD2, mBD-3 and CRAMP were examined in PMNs after HIF-1α inhibition and exposure to *P. aeruginosa* (Fig. 9A–C). Results were similar to those obtained from the in vivo model; all three peptides were reduced after 17-DMAG treatment (in a dose-dependent response) when compared to positive controls (bacteria only, no DMAG). The highest concentration of DMAG exhibited the strongest ability to inhibit production of all three peptides. Overall, similar trends also were observed for cells treated with GM-CSF. In light of these data, antimicrobial peptide expression appears to be a significant variable influencing bacterial load (Table S1).

**Figure 10:**
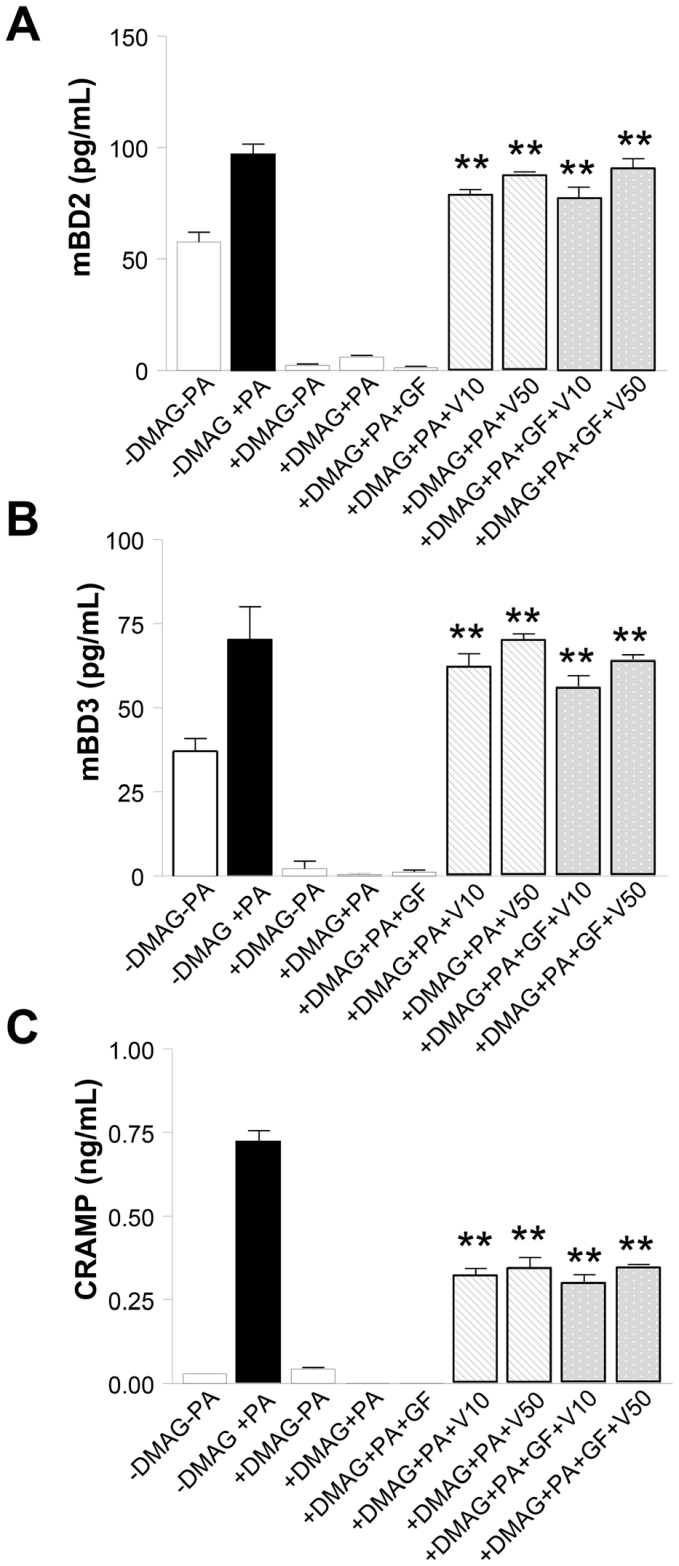
In vitro expression of antimicrobial peptides after HIF-1α inhibition and growth factor treatment. PMN were incubated +/− DMAG treatment (10 µM) for 18 hours, then treated (4 hours) with either VEGF alone (10 and 50 µg/mL), a growth factor (GF) cocktail (HGF, FGF and EGF) or a combination of VEGF+GF cocktail. After challenge with *P. aeruginosa* (2.5∶1 ratio of bacteria∶cells), antimicrobial protein levels from supernatants were determined by ELISA. mBD2 (A), mBD3 (B) and CRAMP (C) were significantly up-regulated after VEGF treatment alone and following GF cocktail+VEGF treatment. ** *P*<0.01.

We have previously demonstrated that growth factors contribute to corneal defense through antimicrobial peptide production [45]. Studies also have demonstrated that both VEGF and HGF are released by activated PMNs [46], [47]; while HIF-1α has been shown to activate VEGF [48], [49]. Therefore, to explore the mechanism by which HIF-1α modulates antimicrobial peptide expression, we examined whether it involved the growth factor pathway. HIF-1α was inhibited in peritoneal-elicited PMN using DMAG at 10 µM, the most effective concentration as determined above. Then, different growth factor paradigms were used to ‘rescue’ the production of antimicrobial peptides after *P. aeruginosa* exposure. Addition of VEGF (at both concentrations tested) resulted in a significant increase in protein expression of all three antimicrobial peptides (mBD-2, mBD-3 and CRAMP) when compared to DMAG only (Fig. 10A–C); in fact, levels of mBD-2 and mBD-3 were similar to those observed in the untreated (no DMAG) cells after bacterial challenge. In contrast, the growth factor cocktail alone had no effect on antimicrobial peptide expression. But when paired with VEGF, levels were significantly up-regulated, but did not differ from levels for VEGF alone. Not only do these data suggest that VEGF is a direct modulator of antimicrobial peptides, but that HGF, KGF and EGF are up-stream of the HIF-1α pathway.

In conclusion, inflammation is an inherent component of the immune response. While it is essential to containing infection, resolution of inflammation and restoration of immune homeostasis is equally critical to the host. When left unregulated, inflammation is as detrimental as the infectious agent itself. In particular for the eye, where the visual axis beginning with light transmission through the cornea, and must be protected from the effects of prolonged cell-mediated inflammation. Therefore, it remains critical to identify and better understand mechanisms of immune regulation in this tissue. HIF-1α has been implicated in the regulation of several important myeloid cell functions, including regulation of metabolism, migration, bactericidal potency and phagocytosis using skin infection models [13], zebrafish embryos [50] , models of sepsis [31] and in vitro analyses [13], [15]. Subsequently, HIF-1α has been suggested as a master regulator of innate host defenses [51]. However, the current study is the first to examine both in vivo and in vitro how HIF-1α regulates PMN function using a well-established keratitis model. We have demonstrated that HIF-1α contributes to the resistant phenotype of BALB/c mice through inflammatory cell activation, enhanced antimicrobial peptide production and subsequent bacterial killing, while regulating T cell infiltration and cytokine production, which would otherwise delay resolution and induce susceptibility to *P. aeruginosa*-induced keratitis. How these data will lead to rational development of alternate therapeutics is a challenge. Nonetheless, establishing the central role of this molecule in keratitis is a solid beginning and provides several targets (including VEGF) to further test in experimental treatment paradigms.

## Materials and Methods

### Experimental animal protocol

Eight-week-old female BALB/c mice (The Jackson Laboratory, Bar Harbor, ME) were anesthetized using ethyl ether and placed beneath a stereoscopic microscope (×40 magnification). The left cornea was wounded by making three 1-mm incisions using a sterile 25^5/8^-gauge needle. A 5-µl aliquot containing 1×10^6^ CFU of *P. aeruginosa* ATCC strain 19660, prepared as described before [23], was topically applied to the wounded corneal surface. Eyes were examined daily to monitor disease response. Corneal disease was graded and recorded for each mouse after infection using an established scale [52] for statistical comparison of disease severity at 1, 3 and 5 days post-infection (p.i.). Slit lamp photographs were taken at 5 days p.i. to illustrate the disease response. Corneal perforation was used as the basis for the final time point, which typically occurred at 5 days p.i. in the experimental group of mice. For all in vivo experiments, n = 5 animals/treatment group/time point were used unless otherwise specified. All animals were treated in a manner approved by Wayne State University Institutional Animal Care and Use committee (Approved protocols identification numbers: A-05-02-11 and A-09-03-12) and all work also was in compliance with the Association for Research in Vision and Ophthalmology Statement for the Use of Animals in Ophthalmic and Vision Research. Animals were monitored daily and if they exhibited signs of distress or pain were removed from the study.

### HIF-1α inhibition

For in vivo experiments, inhibition of HIF-1α was achieved using two separate methods: 1) siRNA treatment and 2) drug-induced inhibition. Use of siRNA has been described in previous publications from our laboratory [34], [35]. For these studies, the left eye of each animal received a subconjunctival injection (5 µL) containing 8 µM of siRNA_HIF-1α_ or a non-targeting scrambled sequence (negative control) (Santa Cruz Biotechnology) one day before infection. Subsequently, topical treatment (5 µL/eye/treatment containing 4 µM siRNA or scrambled sequence) was carried out on the day of infection (1×) and at 1 day p.i. (2×). The efficacy and specificity of HIF-1α silencing was confirmed at both the mRNA and protein levels.

The second, independent method of inhibition involved the water-soluble Hsp90 inhibitor, 17-(dimethylaminoethylamino)-17-demethoxygeldanamycin (17-DMAG) (Invivogen), which has been shown to inhibit HIF-1α [53], [54]. 17-DMAG was injected IP (25 mg/kg) daily beginning one day before infection. This dose was chosen based on the results from our preliminary studies, which showed that higher doses were not tolerated by the animals.

### Neutrophil isolation and treatment

Peritoneal PMN from BALB/c mice were harvested as previously described [55], [56] with slight modification. Briefly, mice received an IP injection (1.0 mL) of a 9% casein solution (Difco, Detroit, MI) 27 hours before cell harvest, followed by a second injection 24 hours later. PMN were collected by peritoneal lavage 3 hours after the second injection. Cells were washed (200× g for 10 min) 3×, then isolated using a Percoll gradient (100,000× g for 20 min). Cell viability (>95%) and purity (>90%) was determined, and then cells were resuspended in RPMI 1640 supplemented with 10% FCS (Invitrogen) ± GM-CSF (1 nmol/L) and plated at various concentrations according to each in vitro assay performed as described below. Cells were incubated at 37°C (5% CO2).

For select in vitro assays, cells were incubated with 17-DMAG (0.1–10 µM) or media only (negative control) for 18 hours at 37°C (5% CO2). The inhibitor was removed and cells were washed and resuspended in media only.

Regarding in vitro growth factor (GF) studies, cells were incubated (4 hours) with 17-DMAG (10 µM) or media only (negative control) as specified above, then treated with either recombinant mouse VEGF 164 (10–50 µg/mL) (R&D Systems), a recombinant mouse GF cocktail (EGF [40 mg/mL], KGF [20 mg/mL] and HGF [40 mg/mL], R&D Systems) [43], or both cocktail and VEGF. The cells were assayed as specified below.

### Real-time RT-PCR

Total RNA was isolated from individual whole corneas for in vivo analysis using RNA-Stat 60 (Tel-Test, Friendswood, TX), according to the manufacturer's recommendations, and quantitated by spectrophotometric determination (260 nm). One microgram of total RNA was reverse transcribed as described before [56]. All primer sets (Table 1) for PCR analyses were designed using PrimerQuest (Integrated DNA Technologies) and semi-quantitative real-time RT-PCR was processed using MyiQ Single Color Real-Time RT-PCR Detection System (Bio-Rad, Hercules, CA). PCR amplification conditions were determined using routine methods [57]. Relative transcription levels were calculated using the relative standard curve method that compares the amount of target normalized to an endogenous reference gene, β-actin. Data are shown as the mean ± SD for relative mRNA levels and represent two individual experiments each with five mice per group per time point.

**Table 1:** Nucleotide sequence of the specific primers used for PCR amplification.

Gene	Nucleotide Sequence	Primer
*β-actin*	5′- GAT TAC TGC TCT GGC TCC TAG C -3′	F
	5′- GAC TCA TCG TAC TCC TGC TTG C -3′	R
*HIF-1α*	5′- GAA ACG ACC ACT GCT AAG GCA -3′	F
	5′- GGC AGA CAG GTT AAG GCT CCT -3′	R
*TNF-α*	5′- ACC CTC ACA CTC AGA TCA TCT T -3′	F
	5′- GGT TGT CTT TGA GAT CCA TGC -3′	R
*IL-1β*	5′- CGC AGC AGC ACA TCA ACA AGA GC -3′	F
	5′- TGT CCT CAT CCT GGA AGG TCC ACG -3′	R
*CXCL2/MIP-2*	5′- TGT CAA TGC CTG AAG ACC CTG CC -3′	F
	5′- AAC TTT TTG ACC GCC CTT GAG AGT GG -3′	R
*β-defensin 2*	5′- TCT CTG CTC TCT GCT GCT GAT ATG C -3′	F
	5′- AGG ACA AAT GGC TCT GAC ACA GTA CC -3′	R
*β-defensin 3*	5′- GGA TCC ATT ACC TTC TGT TTG CAT -3′	F
	5′- TGC TAA AAG CTG CAG GTG GAG -3′	R
*CRAMP*	5′- TCT GTG AGG TTC CGA GTG AAG -3′	F
	5′- GGT GAC TGC CCC CAT ACA C -3′	R

F, forward; R, reverse.

### Immunofluorescent staining

Infected eyes of BALB/c mice after siRNA_HIF-1α_ and scrambled siRNA treatment were enucleated (*n* = 3/group/time) under normal conditions (no infection) and 5 days p.i., rinsed briefly in PBS, embedded in Tissue-Tek OCT compound (Sakura, Torrence, CA), and snap frozen in liquid nitrogen. Ten-micrometer-thick sections were cut and mounted to polylysine-coated glass slides. After overnight incubation at 37°C, slides were fixed; HIF-1α: two minute acetone, CD3: five minute 4% paraformaldehyde/0.01 M sodium periodate/0.05 M lysine in phosphate buffer. The tissue was permeabilized with 0.1% sodium citrate containing 0.1% Triton X (r.t. for 10 min) before the blocking step. For immunostaining, sections were incubated with either goat anti-mouse HIF-1α (1/100, Santa Cruz Biotechnologies) or purified rabbit anti-mouse CD3ε (1/100, Abcam) primary antibodies for 1 hour followed by the correlating secondary antibody, Alexa Fluor 594-conjugated donkey anti-goat (1/1500, Invitrogen) (HIF-1α staining) or staining 546-conjugated donkey anti-rabbit (1/1500, Invitrogen) (pan T cell staining) for an additional hour. Sections were then incubated for 2 min with SYTOX Green nuclear acid stain (1/30000, Invitrogen). All control sections were similarly treated, but the primary antibodies were replaced with similar host IgG, ChromPure goat or rat IgG (Jackson ImmunoResearch Laboratories). Finally, sections were visualized and digital images captured with a Leica TCS SP2 confocal laser scanning microscope (Leica Microsystems).

### ELISA

Protein levels for IL-1β, TNF-α, IL-6, IL-18, CXCL2, mBD2, mBD-3, CRAMP and HIF-1α were tested using ELISA kits (R&D Systems, MyBioSource.com, USCN Life Science Inc.). Corneas from HIF-1α silenced or scrambled (control) BALB/c mice were individually collected (*n* = 5/group/time) at 1 and 5 days p.i. Corneas were homogenized in 0.5 mL of PBS with 0.1% Tween 20. For in vitro experiments, cells and supernatants were collected 2 hours after incubation with *P. aeruginosa* ATCC 19660 or PAO1 ATCC 15692 (as described for the apoptosis assay below). All samples were centrifuged at 5,000× g for 5 min and an aliquot of each supernatant was assayed in duplicate or triplicate per the manufacturer's instruction. The reported sensitivities of these assays is as follows: 0.46–4.80 pg/mL for IL-1β, 1.3–1.8 pg/mL for IL-6, <4.0 pg/mL for IL-10, 23.0 pg/mL for IL-18, 0.36–7.21 pg/mL for TNF-α, <1.5 pg/mL for CXCL2, 0.1 ng/mL for HIF-1α, 0.1 ng/mL for CRAMP, <11.5 pg/mL for mBD2 and <7.8 pg/mL for mBD-3.

### Bacterial load

Corneas from siRNA and scrambled control groups were collected at 1 and 5 days p.i. and the number of viable bacteria was quantitated. Individual corneas were homogenized in sterile 0.9% saline containing 0.25% BSA. Serial 10-fold dilutions of the samples were plated on Pseudomonas isolation agar (Difco) in triplicate and plates were incubated overnight at 37°C. Results are reported as log_10_ number of CFU/cornea, ±SEM.

### Myeloperoxidase (MPO) assay

An MPO assay was used to quantitate PMN number in the cornea of both siRNA_HIF-1α_ and scrambled control treated mice. Corneas were removed at 1 and 5 days p.i. and homogenized in 1.0 mL of 50 mM phosphate buffer (pH 6.0) containing 0.5% hexadecyltrimethyl-ammonium. Samples were freeze-thawed and after centrifugation, 100 µL of the supernatant was added to 2.9 mL of 50 mM phosphate buffer containing *o*-dianisidine dihydrochloride (16.7 mg/100 mL) and hydrogen peroxide (0.0005%). The change in absorbancy (460 nm) was monitored for 5 min at 30 sec intervals. The slope of the line was determined for each sample and used to calculate units of MPO/cornea. One unit of MPO activity is equivalent to ∼2×10^5^ PMN [58].

### Greiss reaction

NO levels were determined by measurement of its stable end product, nitrite, using a Giess reagent (1% sulfanilamide/0.1% naphthylethylene diamine dihydrochloride 12.5% H_3_PO_4_) for siRNA_HIF-1α_ versus scrambled control mice. Briefly, corneas were homogenized in 500 µL of degassed PBS and microcentrifuged at 3500 rpm (5 min). Then, 100 µL of supernatant was added to an equal volume of Griess reagent in triplicate on a 96-well microtiter plate and incubated at room temperature (15 min). Absorbance (570 nm) was measured and nitrite concentrations estimated using a standard curve of sodium nitrite. Data are represented as the mean µM nitrite/cornea ± SEM.

### Phagocytosis assay

GFP-expressing *P. aeruginosa* (ATCC 19660) (a kind gift from Dr. Jeffery Hobden, Louisiana State University, USA) were grown to logarithmic phase in peptone tryptic soy broth (OD_540_ = 1.3–1.8), washed twice, resuspended and diluted in 0.9% normal saline to the desired concentration. Bacteria were added to untreated and treated (17-DMAG) PMN at an inoculum of 2.5 bacteria per cell. Culture plates were then centrifuged (500× g, 10 min) and incubated for 2 hours at 37°C. Cells were washed twice with PBS (500× g, 10 min) and incubated with 0.04% Trypan blue (10 min at 37°C) to verify cell viability and quench fluorescence associated with surface-bound bacteria. Bacterial uptake was quantified using image-based cell counting and fluorescence detection (Cellometer Vision, Nexcelom Bioscience). All samples were counted four times and results are represented as % GFP^+^ cells.

Next, PMN were further evaluated for intracellular detection of GFP^+^ *P. aeruginosa* using a Leica TCS SP2 confocal laser scanning microscope (Leica Microsystems). Cells were incubated for 2 min with SYTOX Orange nuclear acid stain (1/30000, Invitrogen), visualized and then digital images were captured.

### Intracellular killing assay

Intracellular bacterial load was assessed by plate count as previously described with modification [59]. Cells were incubated with *P. aeruginosa* (ATCC 19660) at a ratio of 2.5 bacteria per cell for 1 hour at 37°C. Bacteria were omitted for the negative control. Cells were treated with gentamicin (200 µg/mL) (Sigma) to ensure extracellular and surface-associated bacteria were killed. The contents of each well were centrifuged (500× g, 10 min), then washed twice with Hank's Balanced Salt Solution (HBSS) (Gibco). Cells were lysed with ice cold water, and then plated in triplicate onto Pseudomonas isolation agar plates. Results are represented as CFU/mL of cellular lysate ± SEM.

### Detection of apoptosis

Cells were incubated with *P. aeruginosa* (ATCC 19660) or PAO1 (ATCC 15692) (an invasive strain) at a ratio of 2.5 bacteria per cell for 2 hours at 37°C. Bacteria were omitted for the negative control. Cells were washed in cold phosphate-buffered saline (PBS), then resuspended in an annexin-binding buffer containing propidium iodide (100 µg/mL) and Alexa Fluor 488 annexin V (5 µL/100 µL of cell suspension). Cells were incubated (15 min at r.t.), then immediately analyzed using an image-based cell counting and fluorescence detection instrument (Cellometer Vision, Nexcelom Bioscience) equipped with brightfield and fluorescence channels (excitation/emission peak: 475 nm/535 nm and 525 nm/595 nm). Data are represented as % of viable cells/apoptotic cells/necrotic cells for each treatment group.

### Statistical analysis

The difference in clinical score between two groups at each time point was tested by the Mann-Whitney U test. For all other experiments, an unpaired, two-tailed Student's *t* test (for comparison between two groups) was used. *P*<0.05 was considered to be statistically significant.

## Supporting Information

Figure S1 In vivo expression of HIF-1α after mTOR inhibition. Transcript levels as detected by real-time RT-PCR in rapamycin- versus PBS-treated BALB/c corneas at 1, 3 and 5 days p.i. HIF-1α mRNA was significantly reduced at all time points tested following mTOR inhibition. Data represent two individual experiments each with five mice per group per time point. *P<0.05, ** *P*<0.01, *** *P* < 0.001.
(TIF)

Table S1 Concentration effect of DMAG on bacterial load and antimicrobial production.
(TIF)
