# Supplementary figures and images for: HIF-1α Is Essential for Effective PMN Bacterial Killing, Antimicrobial Peptide Production and Apoptosis in Pseudomonas aeruginosa Keratitis
Source: PLoS Pathog. 2013 Jul 18;9(7):e1003457. doi: 10.1371/journal.ppat.1003457 (PMC3715414; doi:10.1371/journal.ppat.1003457)

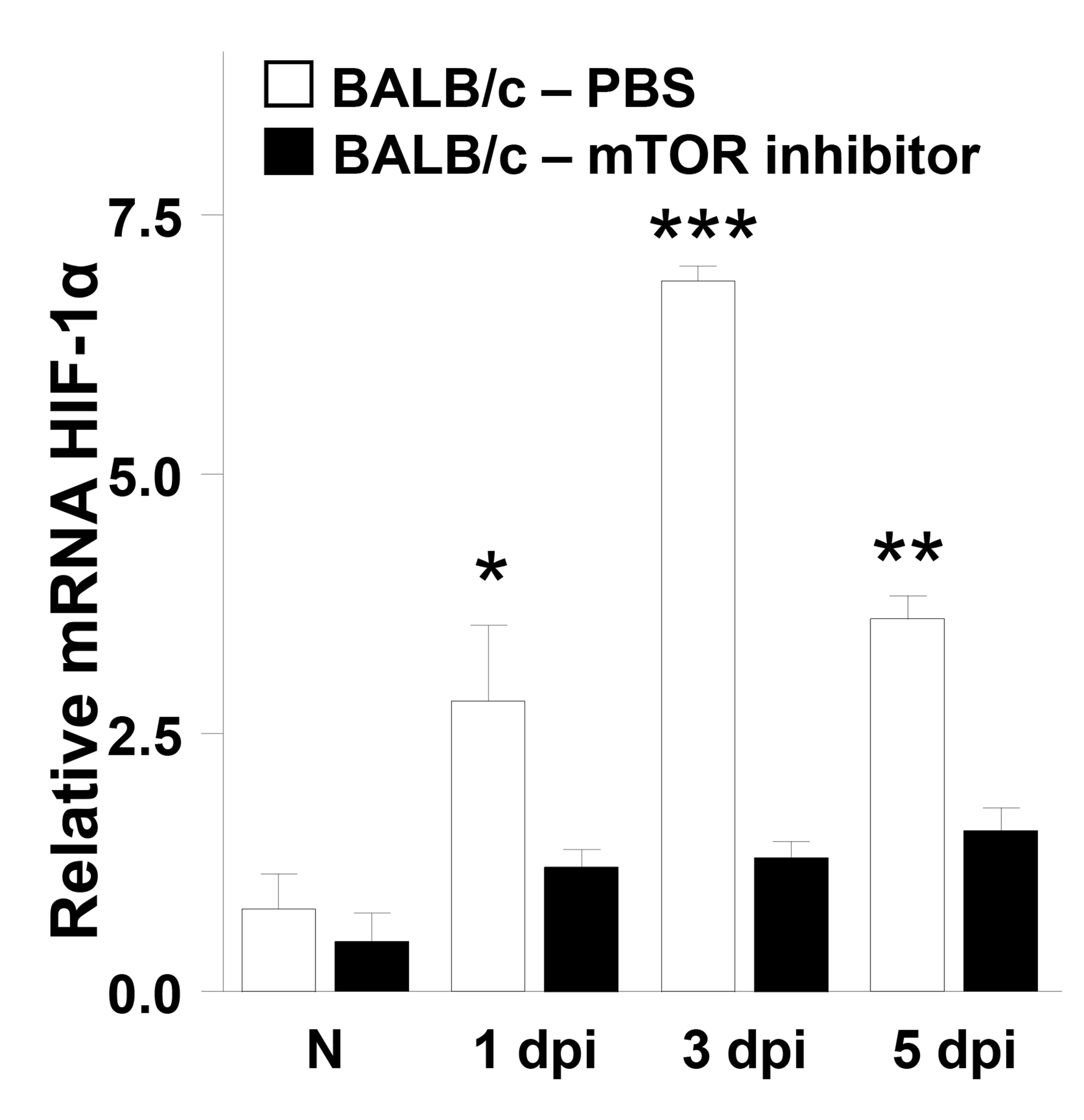

Supplement: Figure S1 — In vivo expression of HIF-1α after mTOR inhibition. Transcript levels as detected by real-time RT-PCR in rapamycin- versus PBS-treated BALB/c corneas at 1, 3 and 5 days p.i. HIF-1α mRNA was significantly reduced at all time points tested following mTOR inhibition. Data represent two individual experiments each with five mice per group per time point. *P<0.05, ** P<0.01, *** P < 0.001. (TIF) [file ppat.1003457.s001.tif]

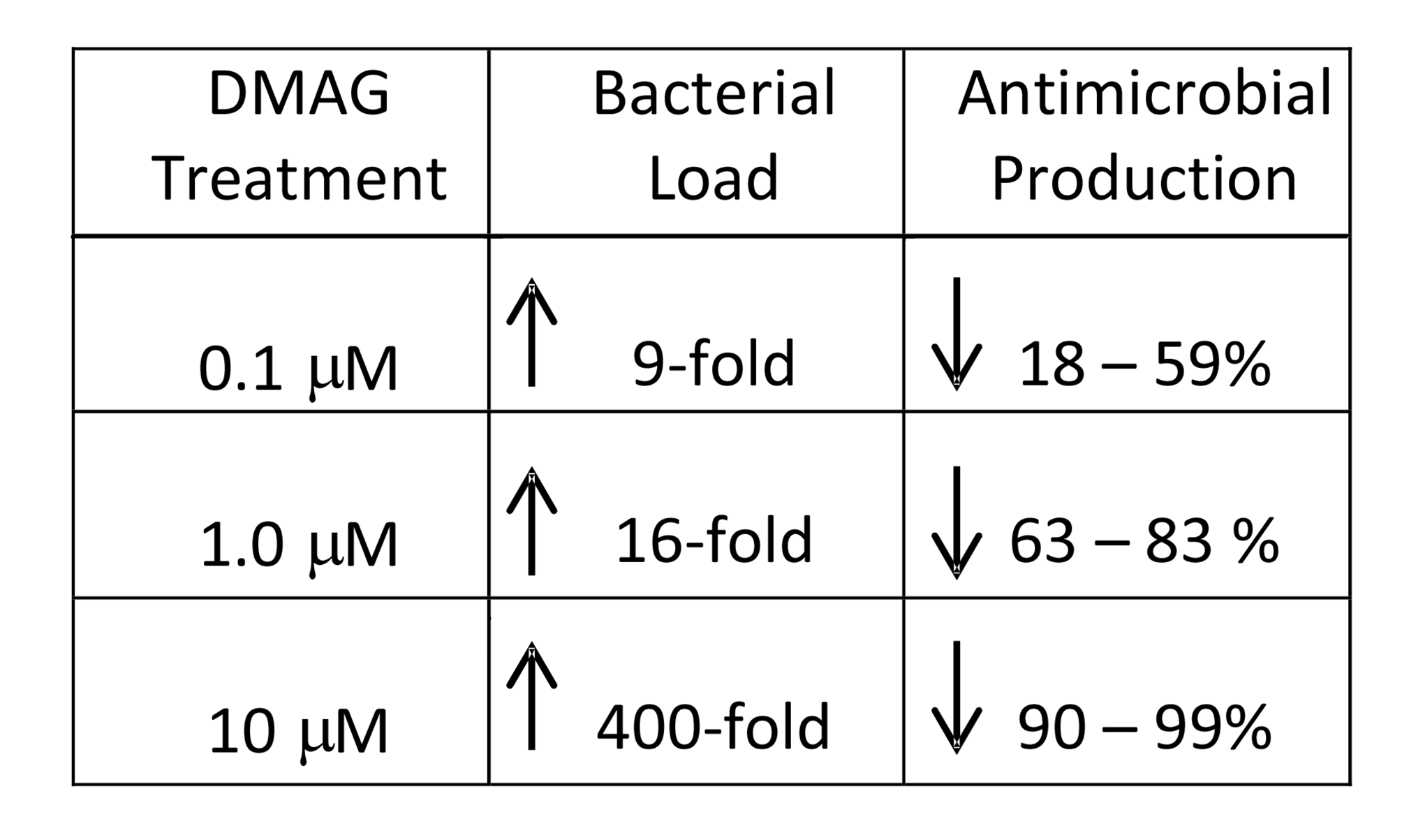

Supplement: Table S1 — Concentration effect of DMAG on bacterial load and antimicrobial production. (TIF) [file ppat.1003457.s002.tif]
